# 1-[Phen­yl(pyridin-2-yl­amino)­meth­yl]-2-naphthol

**DOI:** 10.1107/S1600536810042327

**Published:** 2010-10-23

**Authors:** Jie Xiao, Hong Zhao

**Affiliations:** a School of Chemistry and Chemical Engineering, Southeast University, Nanjing 210096, People’s Republic of China

## Abstract

The title compound, C_22_H_18_N_2_O, was synthesized from naphthalen-2-ol, benzaldehyde and pyridin-2-amine. In the crystal, mol­ecules are linked into centrosymmetric *R*_2_^2^(16) dimers by pairs of O—H⋯N hydrogen bonds. The mol­ecular conformation is stabilized by an N—H⋯O hydrogen bond. The dihedral angle between the naphthylene ring system and the phenyl ring is 72.86 (12)°.

## Related literature

For the application of compounds derived from naphthalen-2-ol in catalytic asymmetric synthesis, see: Szatmari & Fulop (2004 ▶). For related structures, see: Wang & Zhao (2009 ▶); Zhao & Sun (2005 ▶). For graph-set motifs, see: Bernstein *et al.* (1995 ▶).
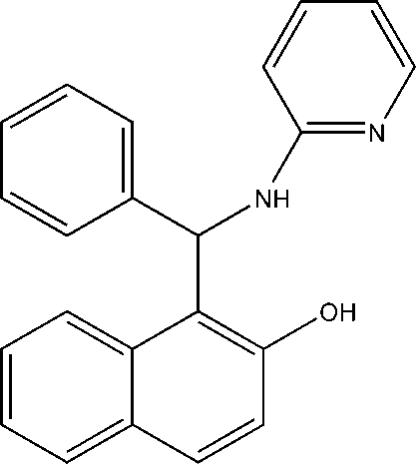


## Experimental

### 

#### Crystal data

C_22_H_18_N_2_O
*M* *_r_* = 326.38
Triclinic, 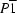

*a* = 7.5841 (10) Å
*b* = 10.1890 (15) Å
*c* = 11.9745 (15) Å
α = 111.00 (3)°
β = 98.64 (5)°
γ = 90.83 (3)°
*V* = 851.7 (2) Å^3^
*Z* = 2
Mo *K*α radiation
μ = 0.08 mm^−1^
*T* = 295 K
0.18 × 0.15 × 0.12 mm

#### Data collection

Rigaku SCXmini diffractometer
Absorption correction: multi-scan (*CrystalClear*; Rigaku, 2005 ▶) *T*_min_ = 0.982, *T*_max_ = 0.990
8701 measured reflections
3841 independent reflections
1655 reflections with *I* > 2σ(*I*)
*R*_int_ = 0.078

#### Refinement

*R*[*F* ^2^ > 2σ(*F* ^2^)] = 0.097
*wR*(*F* ^2^) = 0.218
*S* = 1.10
3841 reflections
230 parameters
H atoms treated by a mixture of independent and constrained refinement
Δρ_max_ = 0.23 e Å^−3^
Δρ_min_ = −0.22 e Å^−3^

### 

Data collection: *CrystalClear* (Rigaku, 2005 ▶); cell refinement: *CrystalClear*; data reduction: *CrystalClear*; program(s) used to solve structure: *SHELXS97* (Sheldrick, 2008 ▶); program(s) used to refine structure: *SHELXL97* (Sheldrick, 2008 ▶); molecular graphics: *SHELXTL/PC* (Sheldrick, 2008 ▶); software used to prepare material for publication: *SHELXTL/PC*.

## Supplementary Material

Crystal structure: contains datablocks I, global. DOI: 10.1107/S1600536810042327/bx2316sup1.cif

Structure factors: contains datablocks I. DOI: 10.1107/S1600536810042327/bx2316Isup2.hkl

Additional supplementary materials:  crystallographic information; 3D view; checkCIF report

## Figures and Tables

**Table 1:** Hydrogen-bond geometry (Å, °)

*D*—H⋯*A*	*D*—H	H⋯*A*	*D*⋯*A*	*D*—H⋯*A*
O1—H1*A*⋯N2^i^	0.82	1.87	2.677 (4)	170
N1—H1*B*⋯O1	0.86	2.35	2.767 (4)	110

Symmetry code: (i) 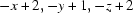.

**Fig. 1.**
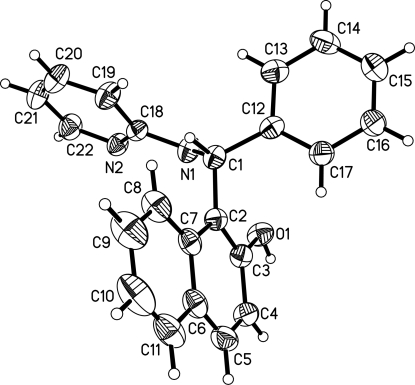
The molecular structure of the title compound, showing the atomic numbering scheme. The displacement ellipsoids are drawn at the 30% probability level.

**Fig. 2.**
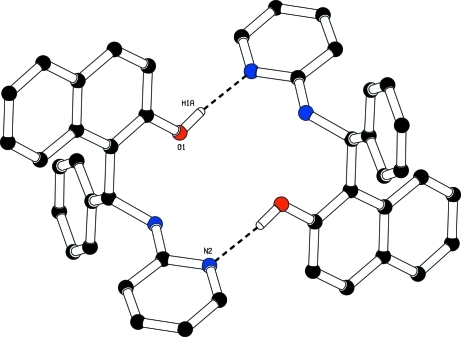
Diagram of the molecules linked into centrosymmetric R22 (16) dimers by a simple O—H···N interaction .The H atoms not involved in hydrogen bonding have been omitted. The atoms no-labelled are related with labelled atoms by symmetry code: (a) -x+2, -y+1, -z+2.

## supplementary crystallographic information

### Comment

Compounds derived from naphthalen-2-ol have been of great interest in organic chemistry due to their application in catalytic asymmetric synthesis (Szatmari & Fulop, 2004; Zhao & Sun, 2005). As an extension of our work on the structural characterization of naphthol compounds (Wang & Zhao, 2009), we report here the structure of the title compound. In the title compound (Fig. 1) bond lengths and angles have normal values. The dihedral angle between the naphthylene ring system and the benzene ring is 72.86 (12)°, and the pyridine ring is 72.61 (11)° respectively. The dihedral angle between benzene ring and the pyridine ring is 74.80 (13)°. In the solid state the molecules are linked into centrosymmetric R^2^_2 _(16) dimers by a simple O—H···N interaction, (Bernstein *et al.*, 1995), (Fig. 2). The molecular conformation is stabilized by one N—H···O hydrogen bonding, Table 1.

### Experimental

A dry 50 ml flask was charged with benzaldehyde (10 mmol), naphthalen-2-ol (10 mmol) and pyridin-2-amine (10 mmol). The mixture was stirred at 100°C for 12 h and then added ethanol (15 ml), after heated under reflux for 1 h, the precipitate was filtrated out and washed with ethanol for 3 times to give the title compound. Colourless crystals suitable for X-ray diffraction were obtained by slow evaporation of a dichloromethane solution.

### Refinement

All H atoms were detected in a difference map, the H-atom bonded to C1 was refined freely, but all other H-atoms were placed in calculated positions and refined using a riding motion approxmation, with C—H = 0.93–0.98 Å, with *U*_iso_(H) = 1.2*U*_eq_(C); O—H = 0.82 Å, with *U*_iso_(H) = 1.5*U*_eq_(O); N—H = 0.86 Å, with *U*_iso_(H) = 1.2*U*_eq_(N).

### Crystal data

**Table:**

C_22_H_18_N_2_O	*Z* = 2
*M**_r_* = 326.38	*F*(000) = 344
Triclinic, *P*1	*D*_x_ = 1.273 Mg m^−^^3^
Hall symbol: -P 1	Mo *K*α radiation, λ = 0.71073 Å
*a* = 7.5841 (10) Å	Cell parameters from 1326 reflections
*b* = 10.1890 (15) Å	θ = 2.7–27.4°
*c* = 11.9745 (15) Å	µ = 0.08 mm^−^^1^
α = 111.00 (3)°	*T* = 295 K
β = 98.64 (5)°	Prism, colourless
γ = 90.83 (3)°	0.18 × 0.15 × 0.12 mm
*V* = 851.7 (2) Å^3^	

### Data collection

**Table:**

Rigaku SCXmini diffractometer	3841 independent reflections
Radiation source: fine-focus sealed tube	1655 reflections with *I* > 2σ(*I*)
graphite	*R*_int_ = 0.078
Detector resolution: 13.6612 pixels mm^-1^	θ_max_ = 27.5°, θ_min_ = 2.7°
CCD_Profile_fitting scans	*h* = −9→9
Absorption correction: multi-scan (*CrystalClear*; Rigaku, 2005)	*k* = −13→13
*T*_min_ = 0.982, *T*_max_ = 0.990	*l* = −15→15
8701 measured reflections	

### Refinement

**Table:**

Refinement on *F*^2^	Primary atom site location: structure-invariant direct methods
Least-squares matrix: full	Secondary atom site location: difference Fourier map
*R*[*F*^2^ > 2σ(*F*^2^)] = 0.097	Hydrogen site location: inferred from neighbouring sites
*wR*(*F*^2^) = 0.218	H atoms treated by a mixture of independent and constrained refinement
*S* = 1.10	*w* = 1/[σ^2^(*F*_o_^2^) + (0.0643*P*)^2^] where *P* = (*F*_o_^2^ + 2*F*_c_^2^)/3
3841 reflections	(Δ/σ)_max_ < 0.001
230 parameters	Δρ_max_ = 0.23 e Å^−^^3^
0 restraints	Δρ_min_ = −0.22 e Å^−^^3^

### Special details

**Table:**

Geometry. All esds (except the esd in the dihedral angle between two l.s. planes) are estimated using the full covariance matrix. The cell esds are taken into account individually in the estimation of esds in distances, angles and torsion angles; correlations between esds in cell parameters are only used when they are defined by crystal symmetry. An approximate (isotropic) treatment of cell esds is used for estimating esds involving l.s. planes.
Refinement. Refinement of F^2^ against ALL reflections. The weighted R-factor wR and goodness of fit S are based on F^2^, conventional R-factors R are based on F, with F set to zero for negative F^2^. The threshold expression of F^2^ > 2sigma(F^2^) is used only for calculating R-factors(gt) etc. and is not relevant to the choice of reflections for refinement. R-factors based on F^2^ are statistically about twice as large as those based on F, and R- factors based on ALL data will be even larger.

### Fractional atomic coordinates and isotropic or equivalent isotropic displacement parameters (Å^2^)

**Table:**

	*x*	*y*	*z*	*U*_iso_*/*U*_eq_	
O1	1.0666 (3)	0.3873 (3)	0.8523 (2)	0.0617 (8)	
H1A	1.1414	0.4523	0.8658	0.093*	
N1	0.7935 (4)	0.2729 (3)	0.9263 (3)	0.0566 (9)	
H1B	0.8964	0.3093	0.9682	0.068*	
N2	0.6664 (4)	0.4120 (3)	1.0857 (3)	0.0530 (8)	
C1	0.7861 (5)	0.1892 (4)	0.7972 (3)	0.0485 (9)	
C2	0.7985 (5)	0.2805 (4)	0.7231 (3)	0.0470 (9)	
C3	0.9439 (5)	0.3761 (4)	0.7531 (3)	0.0520 (10)	
C4	0.9660 (6)	0.4595 (4)	0.6832 (4)	0.0642 (11)	
H4	1.0659	0.5228	0.7041	0.077*	
C5	0.8400 (6)	0.4467 (5)	0.5847 (4)	0.0700 (12)	
H5	0.8559	0.5009	0.5384	0.084*	
C6	0.6862 (6)	0.3526 (4)	0.5520 (3)	0.0604 (11)	
C7	0.6633 (5)	0.2706 (4)	0.6231 (3)	0.0545 (10)	
C8	0.5032 (6)	0.1824 (4)	0.5882 (4)	0.0720 (13)	
H8	0.4822	0.1276	0.6331	0.086*	
C9	0.3793 (7)	0.1749 (5)	0.4913 (5)	0.0960 (17)	
H9	0.2751	0.1165	0.4718	0.115*	
C10	0.4070 (8)	0.2538 (6)	0.4213 (5)	0.0990 (19)	
H10	0.3222	0.2467	0.3542	0.119*	
C11	0.5562 (7)	0.3408 (5)	0.4499 (4)	0.0817 (14)	
H11	0.5740	0.3932	0.4024	0.098*	
C12	0.9204 (5)	0.0761 (4)	0.7796 (3)	0.0488 (9)	
C13	0.9214 (6)	−0.0125 (4)	0.8453 (3)	0.0650 (11)	
H13	0.8439	0.0007	0.9010	0.078*	
C14	1.0357 (7)	−0.1196 (5)	0.8290 (4)	0.0769 (13)	
H14	1.0342	−0.1783	0.8732	0.092*	
C15	1.1510 (6)	−0.1395 (5)	0.7479 (4)	0.0793 (13)	
H15	1.2311	−0.2094	0.7388	0.095*	
C16	1.1481 (6)	−0.0558 (4)	0.6801 (4)	0.0739 (13)	
H16	1.2231	−0.0718	0.6226	0.089*	
C17	1.0346 (5)	0.0526 (4)	0.6962 (3)	0.0607 (11)	
H17	1.0355	0.1096	0.6506	0.073*	
C18	0.6481 (5)	0.2977 (4)	0.9857 (3)	0.0476 (9)	
C19	0.4937 (5)	0.2068 (4)	0.9475 (4)	0.0648 (12)	
H19	0.4829	0.1254	0.8784	0.078*	
C20	0.3584 (6)	0.2405 (5)	1.0143 (4)	0.0775 (13)	
H20	0.2535	0.1820	0.9890	0.093*	
C21	0.3736 (6)	0.3571 (5)	1.1164 (4)	0.0780 (13)	
H21	0.2824	0.3802	1.1625	0.094*	
C22	0.5309 (6)	0.4390 (5)	1.1480 (4)	0.0689 (12)	
H22	0.5443	0.5195	1.2181	0.083*	
H66	0.668 (4)	0.142 (3)	0.776 (3)	0.041 (9)*	

### Atomic displacement parameters (Å^2^)

**Table:**

	*U*^11^	*U*^22^	*U*^33^	*U*^12^	*U*^13^	*U*^23^
O1	0.0546 (17)	0.0668 (18)	0.0604 (17)	−0.0142 (13)	−0.0016 (13)	0.0244 (15)
N1	0.0449 (19)	0.068 (2)	0.0469 (18)	−0.0050 (15)	0.0048 (14)	0.0102 (17)
N2	0.054 (2)	0.053 (2)	0.0499 (18)	0.0025 (15)	0.0178 (15)	0.0124 (17)
C1	0.043 (2)	0.050 (2)	0.046 (2)	−0.0056 (18)	0.0043 (17)	0.0101 (19)
C2	0.054 (2)	0.042 (2)	0.043 (2)	0.0036 (18)	0.0096 (17)	0.0126 (18)
C3	0.050 (2)	0.056 (2)	0.050 (2)	0.0035 (19)	0.0076 (19)	0.019 (2)
C4	0.068 (3)	0.061 (3)	0.070 (3)	0.006 (2)	0.021 (2)	0.028 (2)
C5	0.087 (3)	0.074 (3)	0.063 (3)	0.027 (3)	0.021 (2)	0.038 (3)
C6	0.066 (3)	0.054 (3)	0.052 (2)	0.018 (2)	0.007 (2)	0.010 (2)
C7	0.062 (3)	0.047 (2)	0.048 (2)	0.0171 (19)	0.0106 (19)	0.009 (2)
C8	0.062 (3)	0.061 (3)	0.077 (3)	0.000 (2)	−0.012 (2)	0.016 (2)
C9	0.085 (4)	0.076 (3)	0.102 (4)	−0.002 (3)	−0.030 (3)	0.020 (3)
C10	0.110 (5)	0.081 (4)	0.072 (3)	0.024 (3)	−0.030 (3)	0.005 (3)
C11	0.102 (4)	0.080 (3)	0.058 (3)	0.037 (3)	0.003 (3)	0.022 (3)
C12	0.050 (2)	0.050 (2)	0.041 (2)	−0.0084 (17)	0.0033 (17)	0.0112 (19)
C13	0.078 (3)	0.060 (3)	0.058 (3)	−0.003 (2)	0.012 (2)	0.023 (2)
C14	0.106 (4)	0.056 (3)	0.075 (3)	0.015 (3)	0.015 (3)	0.032 (3)
C15	0.085 (4)	0.064 (3)	0.089 (3)	0.017 (2)	0.022 (3)	0.024 (3)
C16	0.086 (3)	0.053 (3)	0.085 (3)	0.011 (2)	0.033 (3)	0.019 (3)
C17	0.070 (3)	0.054 (3)	0.060 (2)	0.006 (2)	0.019 (2)	0.020 (2)
C18	0.046 (2)	0.051 (2)	0.048 (2)	−0.0019 (17)	0.0073 (17)	0.020 (2)
C19	0.060 (3)	0.064 (3)	0.057 (2)	−0.010 (2)	0.013 (2)	0.006 (2)
C20	0.054 (3)	0.096 (4)	0.083 (3)	−0.010 (2)	0.017 (2)	0.032 (3)
C21	0.063 (3)	0.096 (4)	0.078 (3)	0.009 (3)	0.033 (2)	0.027 (3)
C22	0.072 (3)	0.066 (3)	0.064 (3)	0.009 (2)	0.024 (2)	0.013 (2)

### Geometric parameters (Å, °)

**Table:**

O1—C3	1.363 (4)	C9—H9	0.9300
O1—H1A	0.8194	C10—C11	1.349 (7)
N1—C18	1.378 (4)	C10—H10	0.9300
N1—C1	1.466 (4)	C11—H11	0.9300
N1—H1B	0.8596	C12—C17	1.378 (5)
N2—C18	1.325 (4)	C12—C13	1.393 (5)
N2—C22	1.335 (5)	C13—C14	1.381 (6)
C1—C2	1.508 (5)	C13—H13	0.9300
C1—C12	1.528 (5)	C14—C15	1.366 (6)
C1—H66	0.97 (3)	C14—H14	0.9300
C2—C3	1.376 (5)	C15—C16	1.371 (6)
C2—C7	1.427 (5)	C15—H15	0.9300
C3—C4	1.415 (5)	C16—C17	1.386 (5)
C4—C5	1.367 (5)	C16—H16	0.9300
C4—H4	0.9300	C17—H17	0.9300
C5—C6	1.413 (6)	C18—C19	1.394 (5)
C5—H5	0.9300	C19—C20	1.368 (5)
C6—C7	1.416 (5)	C19—H19	0.9300
C6—C11	1.418 (5)	C20—C21	1.354 (6)
C7—C8	1.415 (5)	C20—H20	0.9300
C8—C9	1.357 (6)	C21—C22	1.368 (6)
C8—H8	0.9300	C21—H21	0.9300
C9—C10	1.388 (7)	C22—H22	0.9300
			
C3—O1—H1A	109.5	C9—C10—H10	119.8
C18—N1—C1	125.0 (3)	C10—C11—C6	120.3 (5)
C18—N1—H1B	117.5	C10—C11—H11	119.9
C1—N1—H1B	117.5	C6—C11—H11	119.9
C18—N2—C22	117.9 (3)	C17—C12—C13	118.2 (4)
N1—C1—C2	112.2 (3)	C17—C12—C1	122.6 (3)
N1—C1—C12	110.8 (3)	C13—C12—C1	119.0 (3)
C2—C1—C12	114.6 (3)	C14—C13—C12	121.1 (4)
N1—C1—H66	102.0 (18)	C14—C13—H13	119.5
C2—C1—H66	109.0 (18)	C12—C13—H13	119.5
C12—C1—H66	107.4 (19)	C15—C14—C13	120.0 (4)
C3—C2—C7	119.2 (3)	C15—C14—H14	120.0
C3—C2—C1	119.0 (3)	C13—C14—H14	120.0
C7—C2—C1	121.7 (3)	C14—C15—C16	119.6 (4)
O1—C3—C2	117.8 (3)	C14—C15—H15	120.2
O1—C3—C4	121.0 (3)	C16—C15—H15	120.2
C2—C3—C4	121.2 (4)	C15—C16—C17	120.8 (4)
C5—C4—C3	119.7 (4)	C15—C16—H16	119.6
C5—C4—H4	120.2	C17—C16—H16	119.6
C3—C4—H4	120.2	C12—C17—C16	120.2 (4)
C4—C5—C6	121.2 (4)	C12—C17—H17	119.9
C4—C5—H5	119.4	C16—C17—H17	119.9
C6—C5—H5	119.4	N2—C18—N1	115.6 (3)
C5—C6—C7	119.0 (4)	N2—C18—C19	121.3 (3)
C5—C6—C11	120.6 (4)	N1—C18—C19	123.1 (4)
C7—C6—C11	120.4 (4)	C20—C19—C18	118.2 (4)
C8—C7—C6	116.1 (4)	C20—C19—H19	120.9
C8—C7—C2	124.3 (4)	C18—C19—H19	120.9
C6—C7—C2	119.6 (4)	C21—C20—C19	121.5 (4)
C9—C8—C7	122.3 (5)	C21—C20—H20	119.2
C9—C8—H8	118.9	C19—C20—H20	119.2
C7—C8—H8	118.9	C20—C21—C22	116.2 (4)
C8—C9—C10	120.5 (5)	C20—C21—H21	121.9
C8—C9—H9	119.8	C22—C21—H21	121.9
C10—C9—H9	119.8	N2—C22—C21	124.8 (4)
C11—C10—C9	120.4 (5)	N2—C22—H22	117.6
C11—C10—H10	119.8	C21—C22—H22	117.6
			
C18—N1—C1—C2	102.8 (4)	C8—C9—C10—C11	−1.1 (8)
C18—N1—C1—C12	−127.8 (4)	C9—C10—C11—C6	−0.1 (8)
N1—C1—C2—C3	56.8 (4)	C5—C6—C11—C10	−178.0 (5)
C12—C1—C2—C3	−70.6 (4)	C7—C6—C11—C10	1.7 (6)
N1—C1—C2—C7	−122.9 (4)	N1—C1—C12—C17	−132.7 (4)
C12—C1—C2—C7	109.6 (4)	C2—C1—C12—C17	−4.6 (5)
C7—C2—C3—O1	177.1 (3)	N1—C1—C12—C13	51.0 (4)
C1—C2—C3—O1	−2.7 (5)	C2—C1—C12—C13	179.1 (3)
C7—C2—C3—C4	−3.2 (5)	C17—C12—C13—C14	1.1 (6)
C1—C2—C3—C4	177.1 (3)	C1—C12—C13—C14	177.5 (4)
O1—C3—C4—C5	−179.5 (3)	C12—C13—C14—C15	0.4 (7)
C2—C3—C4—C5	0.8 (6)	C13—C14—C15—C16	−2.3 (7)
C3—C4—C5—C6	0.8 (6)	C14—C15—C16—C17	2.6 (7)
C4—C5—C6—C7	0.1 (6)	C13—C12—C17—C16	−0.7 (6)
C4—C5—C6—C11	179.8 (4)	C1—C12—C17—C16	−177.0 (3)
C5—C6—C7—C8	177.7 (4)	C15—C16—C17—C12	−1.1 (6)
C11—C6—C7—C8	−2.0 (5)	C22—N2—C18—N1	−178.6 (3)
C5—C6—C7—C2	−2.4 (5)	C22—N2—C18—C19	−1.1 (5)
C11—C6—C7—C2	177.9 (4)	C1—N1—C18—N2	−156.4 (3)
C3—C2—C7—C8	−176.2 (4)	C1—N1—C18—C19	26.2 (6)
C1—C2—C7—C8	3.6 (6)	N2—C18—C19—C20	1.7 (6)
C3—C2—C7—C6	4.0 (5)	N1—C18—C19—C20	179.0 (4)
C1—C2—C7—C6	−176.3 (3)	C18—C19—C20—C21	−1.4 (7)
C6—C7—C8—C9	0.8 (6)	C19—C20—C21—C22	0.4 (7)
C2—C7—C8—C9	−179.1 (4)	C18—N2—C22—C21	0.1 (6)
C7—C8—C9—C10	0.7 (7)	C20—C21—C22—N2	0.3 (7)

### Hydrogen-bond geometry (Å, °)

**Table:**

*D*—H···*A*	*D*—H	H···*A*	*D*···*A*	*D*—H···*A*
O1—H1A···N2^i^	0.82	1.87	2.677 (4)	170
N1—H1B···O1	0.86	2.35	2.767 (4)	110

Symmetry codes: (i) −*x*+2, −*y*+1, −*z*+2.
